# Preschool child growth attainment and velocity during an agriculture intervention in rural Panama may be diminished by soil-transmitted helminths

**DOI:** 10.3389/fpubh.2023.1122528

**Published:** 2023-09-27

**Authors:** Rachel J. Krause, Marilyn E. Scott, Odalis T. Sinisterra, Kristine G. Koski

**Affiliations:** ^1^Department of Science, Canadian Mennonite University, Winnipeg, MB, Canada; ^2^Institute of Parasitology, McGill University, Montreal, QC, Canada; ^3^Department of Nutrition, Ministry of Health, Panama City, Panama; ^4^School of Human Nutrition, McGill University, Montreal, QC, Canada

**Keywords:** preschool children, intestinal infections, hookworm, *Ascaris*, malnutrition, stunting, agricultural intervention, Panama

## Abstract

**Background:**

Agricultural interventions are often recommended to address undernutrition in subsistence farming communities. However, intensified agriculture exposure can increase soil transmitted helminth (STH) infections, which are linked with poor child growth. This study examined impacts of the VERASAN public health and agricultural intervention program on preschool child growth attainment (HAZ and WAZ) and relative growth velocity over 7 months [change in height-for-age (∆HAD) and weight-for-age (∆WAD) difference].

**Methods:**

VERASAN was initiated in 15 subsistence farming communities in rural Panama experiencing chronic undernutrition. Activities targeted improved household food security, preschool child diets and growth by intensifying and diversifying household agriculture. Our objectives were to explore the relationship between VERASAN and preschool child growth attainment (HAZ and WAZ) and velocity (∆HAD and ∆WAD) during one agricultural cycle in 238 households. We compared those new to VERASAN with those involved for 1 or 5 years, and identified if agricultural practices, food security, diet diversity and treatment of pre-existing STH infection were associated with growth attainment or velocity.

**Results:**

Prior participation in VERASAN did not directly influence WAZ, HAZ or ΔHAD but VERASAN-related benefits had an indirect influence. ΔHAD was positively associated with VERASAN-associated improvements in diet diversity and food security. HAZ and WAZ during land preparation were positively associated with diet diversity and HAZ with food security during harvest. HAZ was negatively associated with children visiting the agricultural plot, consuming leafy green vegetables and pre-existing hookworm infections. Both agricultural season and STH influenced ΔWAD. Children in VERASAN for 1 or 5 years experienced growth faltering between land preparation and growing season, but not those new to VERASAN. In contrast, between growing and harvest, ∆WAD declined in children new to VERASAN compared to children in VERASAN for longer. ΔWAD from land preparation to harvest was higher with pre-existing *Ascaris* infection whereas it was lower between growing season and harvest for pre-existing hookworm infection.

**Conclusion:**

In a context of preschool child growth faltering, malnutrition and STH infections, improved food security, agricultural production and diet diversity associated with VERASAN were associated with improved growth. In contrast, STH infections were negatively associated with some, but not all, growth outcomes.

## 1. Introduction

Agricultural interventions have become an important tool in eradicating poverty and undernutrition, particularly in poor, rural areas of developing countries that practice some level of subsistence agriculture (1–3). There has been considerable effort to design nutrition-sensitive interventions (4–7) around objectives to improve the supply of nutritious foods in lean seasons of the year (8, 9), and to increase dietary diversity by including a greater variety of nutrient-dense and micronutrient-rich foods (7, 10–14). However, Ruel and Alderman (4) define “nutrition-sensitive” agricultural interventions as those that go beyond providing an adequate diet to addressing underlying causes, including food insecurity, insufficient childcare, gender inequity, and an unhygienic environment.

Child growth has also been shown to be negatively impacted by chronic infection with soil-transmitted helminths (STHs), with infection and malnutrition mutually reinforcing each other (15–17). Co-occurrence of STH infection and undernutrition is well documented in rural developing regions (18). Further, there is evidence that preschool child growth is improved when STH treatment is incorporated in interventions that provide nutritional support (18–20). However, most agricultural interventions have disregarded the impairment that STHs have on preschool child growth, including farming communities where STHs are endemic (10, 21–24). Undetected STH infections may help to explain why recent reviews on the impact of agriculture-based interventions have not shown the growth benefits, especially height-for-age *z*-scores (HAZ), that would be expected to accompany improved nutrition and diet (4, 25–30).

In the province of Veraguas in Panama, approximately 50% of preschool children in the poorest, rural subsistence farming communities experience chronic undernutrition (31). In response, the agricultural intervention VERASAN (“Proyecto para el mejoramiento del consumo y la disponibilidad de alimentos en comunidades de la provincia de Veraguas,” translated as “Project for the improvement of consumption and availability of food in communities in the province of Veraguas”) was implemented in rural subsistence farming communities in Veraguas by the Panama Ministry of Health (MoH) with support from the Japan International Cooperation Agency and in collaboration with the Panama Ministries of Agricultural Development and Education. The targeted goals were to improve food production for household use, household food security and child diets, and ultimately child growth, by intensifying and diversifying household agriculture. Agricultural training occurred primarily in community demonstration gardens where participants met weekly to learn new agricultural techniques and interact with nutritionists and agricultural extensionists (32).

Five years after the program had begun, our team was invited by the MoH to conduct an interim evaluation of VERASAN outcomes. All households practiced subsistence agriculture on plots approximately 0.9 ha in size and located up to 2 h by foot from the home (33). Households grew rice, maize, beans, pigeon peas, cassava, plantains, cucumbers, squash, sweet peppers, and tomatoes, almost exclusively for their own consumption. Most households also raised chickens and a few had pigs (34). As described previously, at the time of the study, households lived in extreme poverty with an average earned monthly *per capita* income less than $6 USD. Very few households were food secure (34). Homes were adobe or concrete block with 1–2 rooms, palm or tin roofs and dirt or concrete floors. Houses either had untreated water piped to a faucet in the yard or relied on river or other unprotected above-ground sources. Most had pit latrines but very few had electricity. Each community had a primary school, and a few communities had a secondary school and/or a health post which was visited by medical staff on a regular but not daily basis (35). We have previously shown that the VERASAN program was positively associated with improved agricultural practices, increased household food production, and improved household food security and aspects of preschool child diets such as greater animal-source foods and vitamin A-rich foods (32), but that intensification of agricultural practices and the associated increased presence of children on the agricultural plots increased STH transmission and infection in preschool children (33).

The goal of the present study was to examine the impacts of the VERASAN program on preschool child growth attainment (HAZ and WAZ) and relative growth velocity over a 7 month period measured as change in height for age difference (∆HAD) and weight for age difference (∆WAD) in the context of the ongoing delivery of public health and agricultural interventions. We posited that preschool child growth would benefit from diversified diets and improved household food security but might be impaired by exposure to other health risks such as inadequate nutritional intake and STH transmission when the preschool child accompanies the caregiver to the household agricultural plot for field work. Our specific objective was to assess whether VERASAN involvement and/or agricultural, environmental, dietary, and STH variables were associated with preschool child growth attainment (HAZ and WAZ) or relative growth velocity (∆HAD and ∆WAD) during one agricultural cycle that included land preparation, growing and harvest seasons.

## 2. Materials and methods

### 2.1. Study design

**Figure 1:**
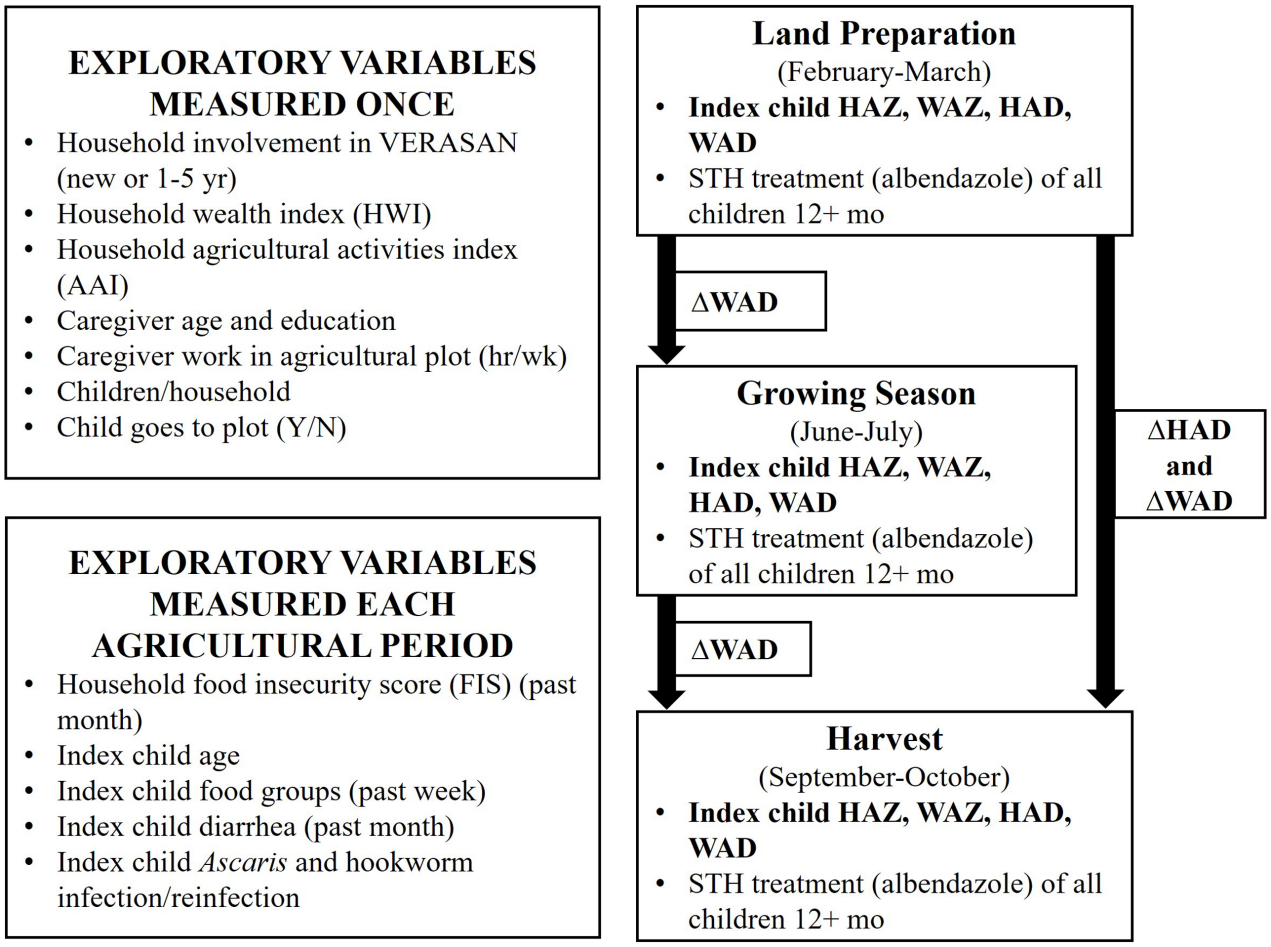
Study design and variables measured over the study during land preparation, growing, and harvest seasons.

This 7-month longitudinal, interim program evaluation followed participating families of the 15 VERASAN communities during one agricultural cycle in 2012. At the start of the evaluation in January 2012, 8 of the communities had been involved in VERASAN for 5 years, 5 for 1 year; and 2 were new to VERASAN. Communities with 1 or 5 years participation did not differ significantly in growth outcomes and therefore were pooled for all analyses (data not shown). Outcomes were measured at 3.5 months intervals in land preparation (February–March), growing season (June–July) and harvest (September–October) periods (Figure 1). The three inclusion criteria for households were: (1) the household practiced subsistence agriculture; (2) at least one member of the household reported participating in VERASAN programming; and (3) at least one child was between the ages of 6 months and 5 years at recruitment. All eligible preschool children in the household were enrolled in the study and one preschool child per household who was not breastfeeding was randomly selected as the index child for data analysis.

### 2.2. Recruitment and ethics protocol

The study protocol was reviewed and approved by the Internal Review Board of the Faculty of Medicine of McGill University, Canada, and the National Research Bioethics Committee of the Gorgas Commemorative Institute for the Study of Health, Panama. Prior to visiting the study communities, permission was obtained from the national and regional directors of the Panama MoH. Then, the lead author and local MoH staff visited all study communities, explained the research, answered questions from community members, and obtained verbal permission to conduct the study. When households were formally recruited into the study, the research was again explained, questions answered, and signed consent was obtained from a representative adult.

### 2.3. Household economic and sociodemographic variables

Demographic and socioeconomic data characteristics relevant for the present analysis are summarized in Table 1. This includes the asset-based household wealth index (HWI) developed previously for these subsistence farming communities (34). We chose to use HWI instead of income because HWI has been shown to be a more reliable measure of longer-term household wealth than irregular monthly income that is easily missed on infrequent questionnaires (36, 37). Our HWI was constructed using principal components analysis of 12 variables including durable assets, home building materials, type of roof, floor and cooking stove, and ownership of a variety of agricultural tools. HWI values averaged 0.78 ± 0.05 and ranged from −0.58 to 2.74 (34, 35).

**Table 1:** Household demographic, caregiver, and index preschool child characteristics during land preparation, growing and harvest periods (values are percentages or means ± standard error^1,2^).

	Land preparation	Growing season	Harvest
Demographic and behavioral variables			
Child age at baseline (mo)	40.4 ± 1.1	–	–
Child sex, % female	50.8	–	–
Caregiver age (year)	33.1 ± 0.6	–	–
Caregiver education (year)	5.0 ± 0.2	–	–
Children ≤12 years in household (#)	3.0 ± 0.1	–	–
Caregiver hours in plot (hours/week)	11.1 ± 1.0	–	–
Child goes to plot (%)	27.6	–	–
Food security and diet			
Food insecurity score (FIS)	7.9 ± 0.6	7.3 ± 0.5	6.6 ± 0.5
Diet diversity score (DDS)	4.7 ± 0.1	5.3 ± 0.1	4.7 ± 0.1
Child ate vitamin A-rich foods (%)	13.9	33.3	23.8
Child ate animal-source foods (%)	88.9	91.3	85.5
Child ate vitamin A-rich leafy greens (%)	4.5	0.7	2.0
Child ate any category of vegetable (%)	57.2	58.0	65.1
Infections			
*Ascaris* baseline prevalence (%)	16.0	–	–
*Ascaris* baseline intensity (epg)	264.6 ± 155.0	–	–
*Ascaris* reinfection prevalence (%)	–	3.8	10.6
*Ascaris* reinfection intensity (epg)	–	127.6 ± 121.5	1421.2 ± 1208.8
Hookworm baseline prevalence (%)	28.2	–	–
Hookworm baseline intensity (epg)	85.2 ± 24.8	–	–
Hookworm reinfection prevalence (%)	–	1.9	3.1
Hookworm reinfection intensity (epg)	–	1.7 ± 1.3	4.0 ± 2.0
Diarrhea prevalence (%)	24.5	36.0	13.9
Diarrhea duration (days in previous 30 days)	0.7 ± 0.1	1.1 ± 0.2	0.4 ± 0.1
Anthropometry			
Height			
Height-for-Age *z*-score (HAZ)	−2.00 ± 0.07	−2.00 ± 0.08	−2.03 ± 0.08
Moderately stunted (%)^3^	37.6	37.0	39.0
Severely stunted (%)^4^	11.8	12.6	11.8
Height-for-Age difference (HAD) (cm)^5^	−7.13 ± 0.31	−7.30 ± 0.34	−8.02 ± 0.34
∆HAD land preparation to harvest (cm) ^6^	–	–	−0.64 ± 0.13
Weight			
Weight-for-Age *z*-score (WAZ)	−1.17 ± 0.08	−1.35 ± 0.08	−1.29 ± 0.08
Overweight (WAZ > 2SD) (%)	1.2	0.0	0.0
Moderately underweight (%)^3^	11.2	13.3	14.7
Severely underweight (%)^4^	5.9	5.9	3.7
Weight-for-Age difference (WAD) (kg)^7^	−1.67 ± 0.14	−2.12 ± 0.15	−2.26 ± 0.14
∆WAD land preparation to harvest (kg)^8^	–	–	−0.58 ± 0.14
∆WAD land preparation to growing (kg)^9^	–	−0.54 ± 0.14	–
∆WAD growing to harvest (kg)^10^	–	–	−0.06 ± 0.10

^1^Sample sizes for variables measured at all three sampling times: land preparation, 170; growing, 135; harvest, 136. ^2^Sample size for variables measured only once, 238. ^3^Z scores <-2SD and > -3SD. ^4^Z scores <-3SD. ^5^Calculated as difference in cm between individual children and median from the WHO reference group. ^6^*n* = 139. ^7^Calculated as difference in kg between individual children and median from the WHO reference group. ^8^*n* = 140. ^9^*n* = 145. ^10^*n* = 125.

A questionnaire administered once during the study provided data on whether the index child accompanied their caregiver to the household’s agricultural plot, how many hours per week the primary caregiver worked in the household’s agricultural plot, and the use of a variety of crop production methods summarized in an agricultural activities index (AAI). The AAI was developed using principal components analysis and included 17 agricultural methods including use of purchased fertilizers and pesticides, methods for improving and conserving soil, and methods for seed saving, planting, and growing. AAI averaged 1.47 ± 0.08 and ranged from 0 to 4.07 (33).

The food insecurity score (FIS) was derived for each sampling period from an experience-based food insecurity scale previously validated for this population that categorized the degree of household-level food insecurity on a scale from 0 (“food secure”) to 42 (“extremely food insecure”) [see details in (34)].

### 2.4. Child diet-derived variables

Data from a semi-quantitative 7-day food frequency recall questionnaire administered for each index child at each sampling period was used to obtain a diet diversity score (DDS) adapted from Arimond et al. (38) based on 11 food categories (grains and starches, legumes, dairy products, eggs, meat and fish, vitamin A-rich leafy green vegetables, orange-fleshed vegetables, vitamin A-rich fruits, vitamin C-rich vegetables, citrus and other vitamin C-rich fruits, and other fruits and vegetables). The final score was the number of categories the child had consumed in the previous 7 days in any quantity (32). In the present analysis, in addition to the DDS we considered all animal-source foods combined, vitamin A-rich fruits and vegetables, vitamin A-rich leafy green vegetables, all vitamin A-source foods combined, and all vegetables combined.

### 2.5. Intestinal infection and treatment

In order to explore whether VERASAN-associated changes in growth were associated with initial STH infection and reinfection, fecal samples were collected at each sampling period for prevalence and intensity, after which all children over 12 months were treated with albendazole according to WHO guidelines to clear *Ascaris* and hookworm (35). Children infected with hookworm also received a further three-day course of albendazole to ensure clearance of this infection (39). This allowed us to measure reinfection during the growth and harvest periods. Also, at each sample collection, caregivers were asked how many days in the previous month each child had experienced diarrhea.

### 2.6. Anthropometry

Given the debate in the literature regarding indices for child catch-up growth (40, 41), both growth attainment and relative growth velocity were used as outcome variables. *Z*-scores are appropriate for cross-sectional analyses (41) but may underestimate growth faltering or improvement over time because the observed height or weight is divided by standard deviations from the WHO global growth standard (42) that increase with age (41, 43, 44). This is corrected by measuring the change in absolute height-for-age difference (∆HAD) and weight-for-age difference (∆WAD) over time (41, 43, 44).

The primary outcome variables were preschool child height and weight, which were used to calculate HAZ and WAZ for each sampling period as well as ∆HAD and ∆WAD over the whole 7-month study (land preparation to harvest), as well as ∆WAD from land preparation to growing season, and from growing season to harvest.

Weight and height/length of each index child were measured during each interval. Weight was measured to the nearest 0.5 kg using a portable anthropometry scale (Seca 750; Seca). Height of children older than 24 months was measured to the nearest 0.1 cm using a portable stadiometer (Seca 214; Seca). Length of younger children was measured to the nearest 0.5 cm using an infant measuring board (Seca 210; Seca). For simplicity, length and height are referred to as “height.”

Growth attainment was calculated as height-for-age *z*-scores (HAZ) and weight-for-age z-scores (WAZ) calculated according to the WHO Child Growth Standards (42), using WHO Anthro software (version 3.2.2) for children 6–60 months and WHO AnthroPlus software (version 1.0.4) for children >60 months. Children with *z*-scores between <-2SD and ≥-3SD were categorized as moderately stunted or underweight, and those with *z*-scores <-3SD were categorized as severely stunted or underweight.

The absolute difference in height (HAD) or weight (WAD) of the index child compared with age- and sex-specific median height or weight from WHO growth standards was calculated at each sampling period. For relative growth velocities, ΔHAD and ∆WAD were calculated over the duration of the study and ΔWAD was also calculated from land preparation to growing and from growing to harvest. Positive ∆HAD and ∆WAD were interpreted as catch-up growth and negative values as further growth faltering.

### 2.7. Data analysis

All statistical analyses were carried out using SAS version 9.3 (SAS Institute Inc., Cary, NC, USA) and statistical significance was set at *p*-values <0.05 unless otherwise noted. All analyses were done on the index child as described above.

Stepwise multiple linear regression analysis was used to explore variables associated with growth attainment (HAZ and WAZ) during land preparation and harvest. All initial stepwise models included socio-demographic and basic household data (child age and sex, caregiver age and years of education, number of children ≤12 years in the household, water piped to the yard, HWI), and our variables of interest: whether the household was new to VERASAN, agricultural variables [AAI, the caregiver worked in the agriculture plot (hr/wk), whether the child went to the agriculture plot with the caregiver] infection variables (presence of *Ascaris* and hookworm at land preparation, *Ascaris* reinfection measured at harvest, presence and days/month of diarrhea), and household food insecurity (FIS), and diet-derived variables (DDS, consumption of vegetables, vitamin A-rich fruits and vegetables, vitamin A-rich leafy green vegetables, and animal-source foods). Independent variables collected during the agricultural period that matched the model were used. However, as HAZ was expected to reflect longer-term infection and nutrition status, HAZ at harvest was first correlated with each diet-derived variable and infection variable from each sampling period and the most strongly correlated variables overall were then included in the initial model. From the initial stepwise models of HAZ and WAZ, independent variables with *p* < 0.3 were selected, and variables with *p* < 0.15 were retained in the final model. Variables were considered non-collinear for variance inflation factors <10 and tolerances >0.10.

In order to understand the underlying growth profile, the relationship of HAD and WAD with child sex and age category was examined using two-way ANOVAs with data from all children at the beginning of the study (land preparation). Growth velocity variables were then explored using three statistical approaches. One-way ANOVA was used to determine if ∆HAD and ∆WAD were associated with prior experience with VERASAN, inclusion of specific items in the child’s diet, and infection with *Ascaris* and hookworm at the beginning of the study (during land preparation). Linear regression analysis was used to explore the relationship of ∆HAD and ∆WAD with FIS and DDS. Logistic regression was used to assess the effects of FIS on diet components and one-way ANOVA was used to assess the effects of being new to VERASAN and eating leafy green vegetables on number of days of diarrhea. Finally, two-way ANOVA was used to explore the impact of measurement interval (land preparation to growing, growing to harvest) and prior involvement with VERASAN on growth velocity as represented by ∆WAD.

## 3. Results

### 3.1. Study participants

**Figure 2:**
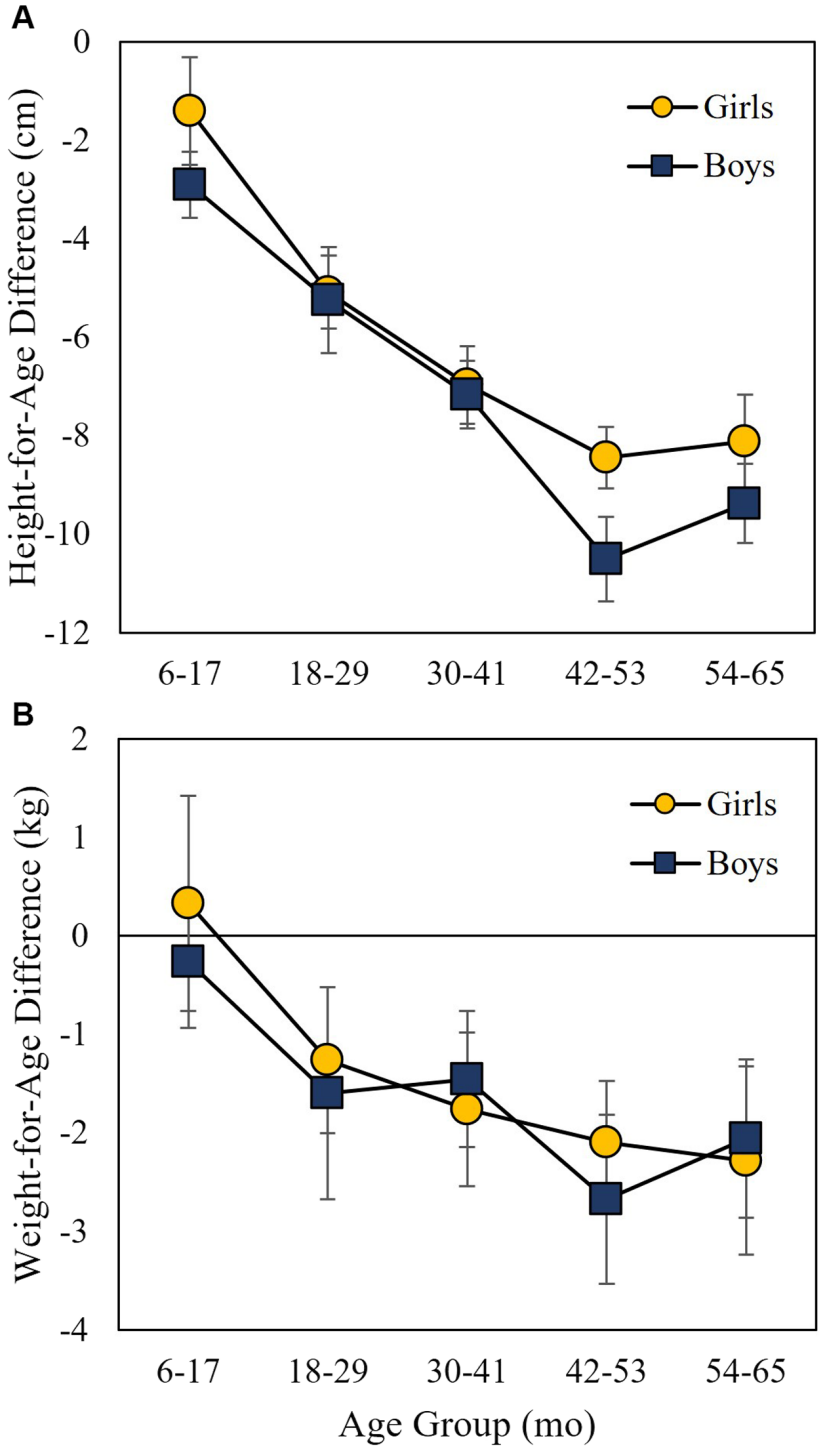
**(A)** Height-for-age difference (HAD) and **(B)** weight-for-age difference (WAD) for index children age 6 months to 5 years by age category and child sex during land preparation.

A total of 238 households were recruited. The 27 households lost to follow-up did not differ from other participating households in socio-demographic, child anthropometry, infection characteristics, AAI or frequency of children visiting the agricultural plot (data not shown). A summary of descriptive data relevant to this study is presented in Table 1. The average age of the 211 index children was 40.4 ± 1.1 months and 50.8% were girls. Almost 50% of index children were stunted (47.1–49.1% depending on sampling period) and underweight was less common (16.2–20.7%) (Table 1). Very few children were overweight. This was reinforced by HAD and WAD (Table 1). Child age category was significantly associated with both HAD and WAD (Figure 2), with older children having greater height and weight deficits relative to the WHO reference data (HAD: *F*_4,190_ = 20.94, *p* < 0.0001; WAD: *F*_4,190_ = 7.56, *p* < 0.0001). Child sex was not associated with HAD or WAD and there was no interaction (Figure 2).

At baseline, 28% of children were infected with hookworm and 16.2% were infected with *Ascaris.* Reinfection prevalence following anthelmintic treatment was very low for both infections between land preparation and growing (*Ascaris,* 3.8%; hookworm, 1.9%) and from growing to harvest (*Ascaris*, 10.6%; hookworm, 3.1%), and intensities were extremely low (Table 1). Diarrhea was common but of very short duration (Table 1) and was higher in children from households with longer exposure to VERASAN (prevalence: 26.7% vs. 9.3%, *X*^2^ = 8.64, *p* = 0.003; duration: 2.5 days/month vs. 0.8 days/month, *F* = 12.6, *p* = 0.0005) although only during the growing season and not during land preparation or harvest.

Children from households with higher food insecurity (higher FIS) were more likely to have consumed leafy green vegetables [OR (95% CI) = 1.12 (1.05, 1.19), *p* = 0.0006] but less likely to have consumed animal-source foods [OR (95% CI) = 0.94 (0.90, 0.98), *p* = 0.005], all measured during land preparation. Children who had consumed vitamin A-rich leafy green vegetables during land preparation experienced significantly more days of diarrhea in the same season (*F* = 10.07, *p* = 0.0018).

### 3.2. HAZ

HAZ was explored in multiple regression models during land preparation and harvest. During land preparation, HAZ was positively associated with diet diversity (DDS) and negatively associated with the child accompanying their caregiver to the household agricultural plot, initial infection with hookworm, and child having eaten leafy green vegetables (Table 2). During harvest, HAZ was negatively associated with being from a household with more children, the child going to the agricultural plot, presence of hookworm at the beginning of the study, higher household food insecurity, and the child eating leafy green vegetables (Table 2). Being new to VERASAN did not enter the multiple regression models. Variables with *p* > 0.15 that were not included in the final models are listed in Table 2.

**Table 2:** Multiple regression models^1^ of HAZ of index children during land preparation (*n* = 107) and harvest (*n* = 103) periods of the study.

	Land preparation		Harvest	
	*β*	*p*	*β*	*p*
Overall model	*R*^2^ = 0.2817	<0.0001	*R*^2^ = 0.2542	<0.0001
Children in household (#)	−0.096	0.0916	−0.129	0.0266
Child went to agriculture plot (Yes)	−0.470	0.0044	−0.443	0.0138
Hookworm infection (Yes)^2^	−0.326	0.0457	−0.396	0.0263
Food Insecurity Score^2^	NI^3^		−0.030	0.0099
Dietary Diversity Score^2^	0.122	0.0278	NI	
Child ate leafy green vegetables (Yes)^2^	−0.916	0.0488	−1.032	0.0200

^1^Variables initially included in the model that did not emerge in any of the final models at *p* < 0.015: household was new to *VERASAN*, child age and sex, caregiver age, household wealth index (HWI), water piped to the yard, number of hours/week. the caregiver worked in the agriculture plot, agricultural activity index reflecting diversity of agricultural methods used, *Ascaris,* number of days of diarrhea in the previous 30 days, consumption of vegetables, vitamin A-rich fruits and vegetables, and animal-source foods. ^2^Measured during land preparation (beginning of the study). ^3^NI, not included in final model (*p* > 0.15).

### 3.3. WAZ

Multiple regression models for WAZ were also constructed during land preparation and harvest. In the land preparation model, WAZ was positively associated with diet diversity (DDS) and negatively associated with consumption of animal-source foods (Table 3). During the harvest, WAZ was positively associated with years of caregiver education and HWI and negatively associated with presence of hookworm at the beginning of the study (Table 3). Being new to VERASAN entered the multiple regression model for harvest but was not significant (*p* = 0.1268). Variables with *p* > 0.15 that were not included in the final models are listed in Table 3.

**Table 3:** Multiple regression models of WAZ^1^ of index children during land preparation (*n* = 107) and harvest (*n* = 98) periods of the study.

	Land preparation		Harvest	
	*β*	*p*	*β*	*p*
Overall model	*R^2^* = 0.1059	0.0184	*R^2^* = 0.2368	0.0005
Household new in VERASAN	NI^5^		0.448	0.1268
Child age (month)	NI		−0.009	0.1235
Caregiver education (year)	0.079	0.0659	0.128	0.0030
Household wealth index	0.233	0.1212	0.273	0.0465
*Ascaris* reinfection (Yes)^3^	NA^6^		0.525	0.1070
Hookworm infection (Yes)^2^	NI		−0.444	0.0332
Diet Diversity Score^4^	0.192	0.0217	NI	
Child ate vegetables (Yes)^4^	NI		0.289	0.1398
Child ate animal-source foods (Yes)^4^	−0.920	0.0208	NI	

^1^Variables initially included in the model that did not emerge in any of the final models at *p* < 0.015: household was new to *VERASAN*, child sex, caregiver age, number of children ≤12 years, water piped to the yard, food insecurity score, number of hours/week, the caregiver worked in the agriculture plot, agricultural activity index reflecting diversity of agricultural methods used, diarrhea (Y/N and number of days in previous month), whether the child went to the agricultural plot, consumption of vitamin A-rich fruits and vegetables, and consumption of leafy green vegetables. ^2^Measured during land preparation. ^3^Measured during harvest (end of the study). ^4^Measured in same season as modeled WAZ. ^5^NI, not included in final model (*p* > 0.15). ^6^NA, not applicable in the season for which the outcome variable was measured.

### 3.4. ∆HAD

**Figure 3:**
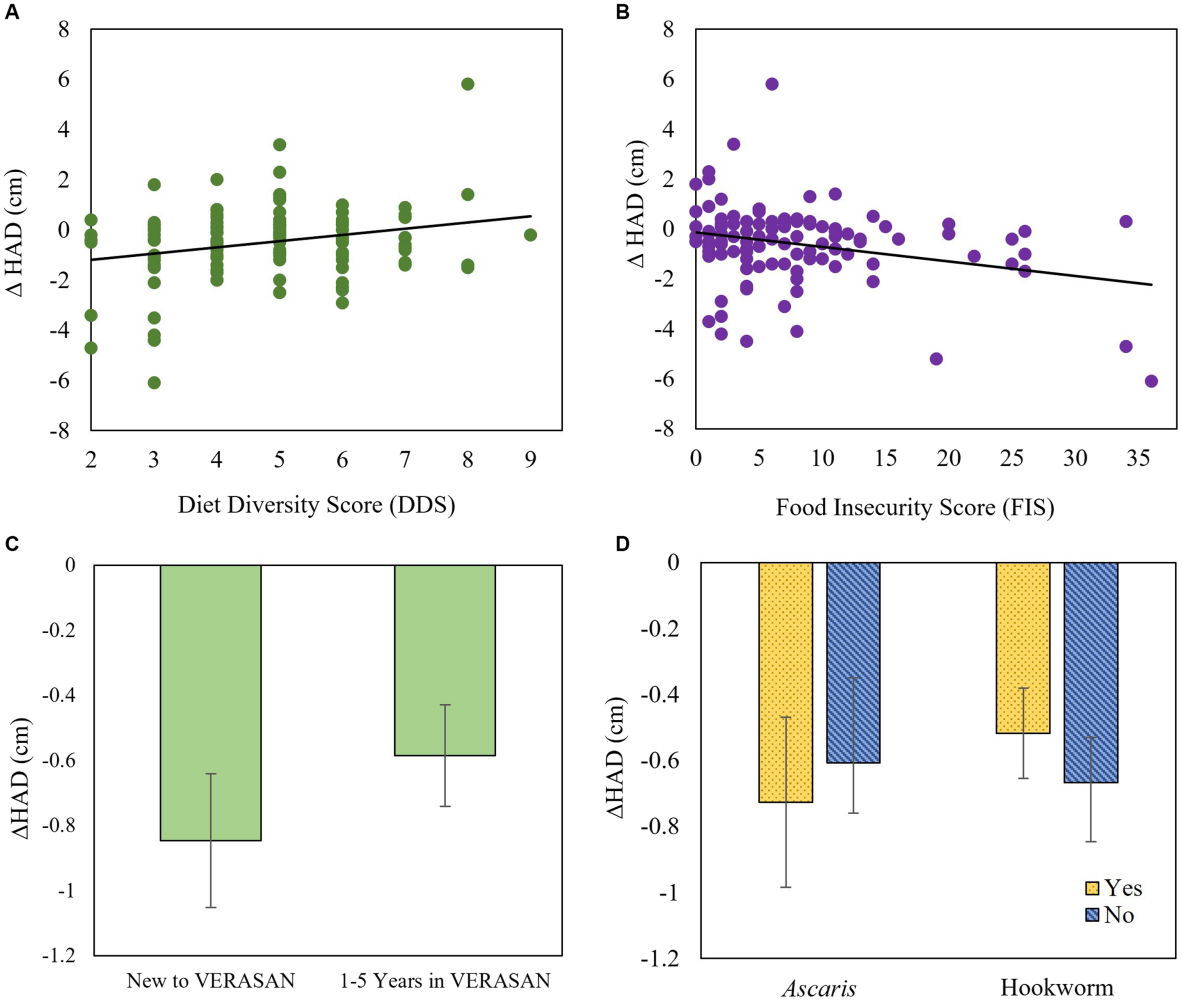
Change in height-for-age difference (∆HAD; kg) for index children age 6 months to 5 years compared to the WHO growth standard during land preparation to harvest (whole study) by: **(A)** new or 1–5 years in the *VERASAN* intervention, **(B)** baseline *Ascaris* and hookworm infections, **(C)** diet diversity score [DDS; measured during harvest; *β* (95% CI) = 0.250 (0.007, 0.423), *p* = 0.005], and **(D)** food insecurity score [FIS; measured during the growing season; *β* (95% CI) = −0.058 (−0.097, −0.019), *p* = 0.004].

Linear regression analyses revealed that ∆HAD from land preparation to harvest increased with a more diverse diet (Figure 3A) and decreased with greater household food insecurity (Figure 3B) but did not differ between those new to VERASAN and those involved for 1 to 5 years (Figure 3C). In addition, ∆HAD was unaffected by presence of *Ascaris* or hookworm at the beginning of the study (Figure 3D), or by consumption of vitamin A-source or animal-source foods (data not shown).

### 3.5. ∆WAD

**Figure 4:**
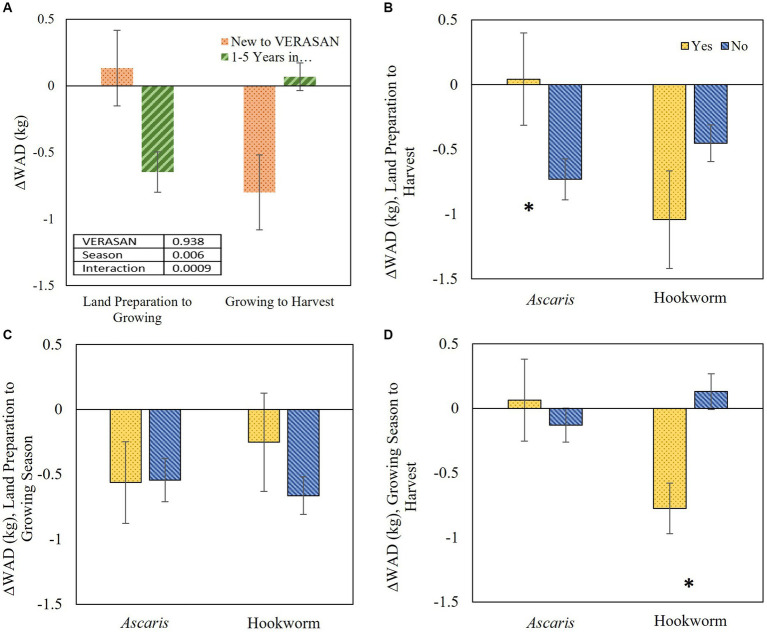
Change in weight-for-age difference (∆WAD; kg) for index children age 6 months to 5 years compared to the WHO growth standard by: **(A)** being new to *VERASAN* in the first half of the study (land preparation to growing season) and the second half of the study (growing season to harvest); and baseline *Ascaris* and hookworm infections during **(B)** land preparation to harvest (whole study), **(C)** land preparation to growing season (first half of the study), and **(D)** growing season to harvest (second half of the study). Significant differences indicated with * (*p* < 0.05).

Although ∆WAD between land preparation and harvest did not differ between those new to VERASAN and those with longer involvement, there was a highly significant interaction between VERASAN involvement and the two sampling intervals (*p* = 0.0009) reflecting the difference in direction of association with VERASAN between the sampling windows (Figure 4A). During the land preparation-growing interval, we found a positive ∆WAD (0.13 ± 0.28 kg) in children from households new to VERASAN in contrast to growth faltering (−0.65 ± 0.15 kg) in children in households participating for 1 or 5 years. This pattern was not seen in the growing-harvest interval when children new to VERASAN had a lower ∆WAD (−0.80 ± 0.28 kg) relative to the global growth standard in comparison to children from households with 1 or 5 years in VERASAN, whose ∆WAD was 0.07 ± 0.10 kg.

Presence of both *Ascaris* and hookworm at the beginning of the study affected ∆WAD, a measure of relative growth velocity for weight. Compared with children with no *Ascaris* who showed growth faltering between land preparation and harvest (∆WAD –0.73 ± 0.16 kg), those initially infected with *Ascaris* had a significantly higher ∆WAD (0.04 ± 0.35 kg) (Figure 4B). When separated into the two study intervals, ∆WAD was unaffected by initial STH infection between land preparation and growing season (Figure 4C) but ∆WAD between the growing and harvest periods was significantly lower (−0.78 ± 0.20 kg vs. 0.13 ± 0.14 kg) in children who had been infected with hookworm at the beginning of the study (Figure 4D), indicating growth faltering. ∆WAD was not associated with FIS, DDS, or consumption of vitamin A-source or animal-source foods (data not shown).

## 4. Discussion

Important goals of agricultural interventions in subsistence farming communities are to increase food availability and diet diversity, to reduce household food insecurity and to improve growth of children, especially preschool children who routinely fall outside school feeding programs. Previously we showed that the VERASAN intervention in subsistence farming communities in Panama improved agricultural production, food security and preschool child diets (32, 34) but that STH prevalence in preschool children was positively associated with visiting the agricultural plot and with the hours the caregiver spent per week on the plot (33, 35). This highlighted the likelihood of transmission on the plot, potentially limiting growth (15–17). Our goal was to identify among this set of factors those that were positively or negatively associated with preschool child growth.

Three major findings emerged. First, participation in VERASAN for 1 or 5 years did not directly influence child height (HAZ) or weight (WAZ) or ΔHAD but VERASAN-related outcomes did. The VERASAN-related increase in diet diversity was positively associated with HAZ and WAZ, VERASAN-related reduction in food insecurity was positively associated with HAZ but not WAZ, and the VERASAN-related increase in intake of leafy green vegetables was negatively associated with HAZ. Similarly, ΔHAD was positively associated with the VERASAN-driven improvements in diet diversity and food security. Second, VERASAN increased the time that caregivers spent on their agricultural plots with their preschool children and their presence on the plot was positively associated with hookworm infection and hookworm was negatively associated with WAZ. Third, both agricultural season and STH influenced relative change in weight. Whereas ∆WAD relative to the global growth standard from land preparation to growing was marginally positive in children from households new to VERASAN, children from households in VERASAN for 1 or 5 years experienced growth faltering. However, between growing and harvest, ∆WAD declined in children new to VERASAN in comparison to children participating in VERASAN for 1 to 5 years.

To date, reviews of nutrition-sensitive agricultural interventions have shown limited impacts of interventions on child anthropometry, particularly height gain (4, 25–30). In our study, history of participation in VERASAN did not modify HAZ, WAZ or ∆HAD but for households new to VERASAN, ∆WAD was higher for children between land preparation and growing periods. This might be associated with greater initial support in these new communities from VERASAN staff, through more frequent visits from staff and more resources provided, such as food provided from demonstration gardens that would have supplemented limited stores from the previous harvest (pers. obs.). Between land preparation and growing seasons, children in households with 1 or 5 years in VERASAN showed a negative ∆WAD, which is consistent with lower food availability during the land preparation to growing season. Between growing and harvest, the opposite pattern was seen with those new to the program having much lower ∆WAD, suggesting that these households were not yet experiencing improvements to home agriculture from the intervention. This contrasted with children whose families had been in the intervention for 1 or 5 years, whose weight deficit remained constant relative to the global growth standard, as expected during a more plentiful time of year (growing season to harvest). These findings were not evident using HAZ and WAZ, highlighting the importance of choice of anthropometry indices to capture potential shorter-term as well as longer-term impacts of interventions on child growth, as linear growth depends on adequate weight gain (45, 46). Therefore, measures like WAZ, and in our study ∆WAD, may be expected to respond more quickly to interventions than HAZ and ∆HAD.

### 4.1. Seasonal food insecurity and diet diversity affect child growth

Interventions such as VERASAN would be expected to influence child growth outcomes through, for example, improved household food security and child diet diversity (4, 29, 30, 40) but the ability to observe growth differences can be obscured by seasonal patterns in food availability. Previously we showed that, during land preparation and the growing season, household food security and child diet diversity were higher for households with 1–5 years in VERASAN compared with those that were new, but the association with VERASAN disappeared during harvest when these outcomes, on average, were improved for all households (32). In the present study, we showed that food insecurity was negatively associated only with HAZ and only during harvest, and that diet diversity was positively associated with HAZ and WAZ in land preparation but not during harvest. Thus, seasonality must be taken into account in subsistence contexts in which food availability may fluctuate over the year (47, 48). Furthermore, diet diversity was positively associated with higher growth velocity (∆HAD), highlighting the value of including growth velocity when evaluating agricultural interventions (40).

### 4.2. VERASAN-associated dietary change linked to child growth attainment

Consumption of “vitamin A-rich leafy green vegetables” emerged as a significant predictor of lower HAZ during land preparation and harvest. This negative relationship was unexpected, given that many nutrition-sensitive agricultural interventions in rural, subsistence farming communities in developing countries have focused on increased production and intake of vitamin A-rich foods (10–14, 49). Consumption of leafy green vegetables, which in this setting are gathered in the wild, may be an indicator of lack of household access to preferred foods, given that more food insecure households consumed more leafy green vegetables (32). Similar relationships have been observed in Kenya where wild edible plants were more commonly consumed in households that were more vulnerable to food insecurity (50). In addition, leafy green vegetables are often contaminated with soil- or water-borne pathogens (22, 51, 52), and children who consumed leafy green vegetables experienced more days of diarrhea in the previous month, and diarrhea is negatively associated with linear growth (53–55).

Consumption of nutrient-dense “animal-source foods” emerged as a significant predictor of lower WAZ during land preparation. This may reflect an imbalance between protein and carbohydrates in the season when staples are scarce, as would be the case in many households in these communities during land preparation. Additionally, reliance on animal-source foods may increase during times of scarcity and signal a scarcity of other key food sources, as reported in a study in Mozambique where farming families increased their meat and vegetable consumption during lean periods of the year when maize, the local staple, was scarce (56).

### 4.3. STH infections limit growth attainment and velocity

Previous agricultural interventions have often overlooked STH infection when evaluating intervention effects on child growth (29), even though there are notable examples where failure to detect an impact on growth has been attributed to lack of attention to helminth infections (10, 57). We addressed this shortcoming by measuring STH infection and by repeated albendazole treatment of all preschool children both to clear existing infections and to monitor any reinfection that occurred. Our data indicate that treatment was successful, that minimal reinfection occurred, and that despite repeated treatment, STH infection present at the beginning of the study continued to exert an impact on anthropometric measures up to 7 months after treatment. Consistent with the literature [e.g., (54, 58, 59)], several sociodemographic variables associated with STH infection emerged in at least one of the growth models, specifically child age, maternal education, and household wealth (HWI), as well as child exposure to household agricultural activities (33).

Intestinal nematode infections have been associated with diminished child growth in other populations at risk for malnutrition (60–64) and recovery from any infection-induced pathology can be prolonged in the context of malnutrition (18). We detected a negative association of initial hookworm infection with HAZ both at land preparation and harvest, and with WAZ at harvest. Furthermore, whereas the relative velocity of change in weight (∆WAD) between the growing and harvest was +0.13 kg in children that did not have hookworms at the beginning of the study, ∆WAD was −0.78 kg in those initially infected with hookworm and successfully treated indicating a significant growth faltering. Thus, the association of initial hookworm infection with lower HAZ and WAZ and lower ∆WAD at the end of the study suggests that the pre-existing hookworm infection continued to exert a negative influence on child height and height even 7 months later. The blood feeding and hemorrhaging associated with even low intensity hookworm infection could contribute to anemia and iron deficiency (65) that might persist in undernourished children making catch-up growth more difficult to achieve (18). In contrast to hookworm, initial *Ascaris* infection was positively associated with ∆WAD over the 7 months of the study. Even though light infections with *Ascaris* are not normally pathogenic or a cause of malabsorption (66), initial treatment appeared to provide a growth benefit to the initially infected children relative to initially uninfected children, consistent with studies demonstrating that treatment of *Ascaris* has immediate benefits for growth (16). These observations highlight the importance of considering STH infection as an important modifier of child growth particularly when exploring the impacts of agricultural interventions.

### 4.4. Risks associated with preschool children accompanying their caregiver to the agricultural plot

Interventions such as VERASAN involve a wide range of components that may benefit agricultural production or food security more rapidly than nutritional status or growth. We had hypothesized that the growth benefits resulting from improved diet diversity and food security might have been partially offset by increased exposure of preschool children to infections. Presence of children on the household agricultural plot was associated with lower linear growth attainment (HAZ) in both land preparation and harvest models, perhaps because exposure to intensified subsistence agriculture increased the risk of *Ascaris* and hookworm infections in preschool children and because STH transmission occurred when preschool children accompanied their caregiver who was working on the household agricultural plot (33). However, given the very low reinfection rates, increased presence of children on the plot may have limited child growth through other pathways. Plots were typically some distance from the home, meaning that access to latrines and water was limited. Exposure to unhygienic conditions can result in environmental enteropathy which can lead to growth faltering (67–69). Diarrhea, our only indicator of environmental enteropathy, did not affect growth attainment or velocity, possibly because the reported duration was about 1 day/mo. It is also possible that child feeding patterns may differ when children are in the plot. We did not explore this, but Bolivian women participating in their household’s farming activity lacked the time and foods to feed their children in their agricultural plots, resulting in lower feeding frequency, a less diverse diet, and a lower caloric intake (70). Taken together, children who accompany their caregiver to the agricultural plot are likely exposed to STH transmission and other pathogens and may receive a less nutritious diet, all of which could contribute to lower HAZ.

### 4.5. Strengths and limitations

Our interim program evaluation had three important strengths, each associated with limitations. First, the design of this ongoing multi-sector intervention program reflected the needs of the participants and the logistical requirements of the MoH and its partnering agencies. Our opportunity to evaluate this program allowed us to explore a comprehensive set of variables that potentially influence preschool child growth. However, these strengths limited our ability to follow best practices in intervention research design. All communities were involved with VERASAN, so we were unable to include a true control group. Fortunately, our study coincided with implementation of VERASAN into a new set of communities that we could compare with those involved for 1 to 5 years. Given the scope of data collected, we relied on self-reported qualitative data for diet- and agriculture-related variables, which are less subject to recall bias than quantitative recall data.

Second, to our knowledge, this is the first study to explicitly include STH infections and STH-related infection risks within an agricultural intervention when exploring the impacts of an agricultural intervention on preschool child growth outcomes. However, the study partnership requirement that all children be treated with albendazole throughout the study precluded us from having an untreated comparison group. Low reinfection rates precluded us from directly exploring the impact of reinfection on growth, but we were able to observe the lasting impact of STH infections even after treatment and within this context of low-level transmission.

Third, we were able to explore seasons by incorporating a longitudinal design over one agricultural cycle (land preparation, growing season and harvest), allowing us to evaluate relative growth velocity (∆HAD and ∆WAD) that revealed associations with measured variables that would not have been found using only routine HAZ and WAZ measures. Despite this, the 7-month window was relatively short for detecting changes in growth, particularly linear growth. We were limited to monitoring a single agricultural cycle, precluding us from making conclusions about agricultural seasons.

## 5. Conclusion

Taken together, this interim assessment of the VERASAN initiative provided evidence that the positive impacts on agricultural production, child diet diversity and household food security benefited child growth. It highlights the value of ∆HAD and ∆WAD as sensitive short-term growth indicators. It also indicates that such programs may also have unintended negative consequences for child growth especially if children’s exposure to STH infections and unhygienic conditions is increased. Further research into nutrition-sensitive agricultural interventions should consider potential impacts of agricultural exposures on increased STH infections, as nutrition-sensitive, agricultural interventions that are also “infection-sensitive” may improve child growth.

## Data availability statement

The raw data supporting the conclusions of this article will be made available by the authors, without undue reservation.

## Ethics statement

The studies involving humans were approved by the Internal Review Board of the Faculty of Medicine of McGill University, Canada and the National Research Bioethics Committee of the Gorgas Commemorative Institute for the Study of Health, Panama. The studies were conducted in accordance with the local legislation and institutional requirements. Written informed consent for participation in this study was provided by the participants’ legal guardians/next of kin.

## Author contributions

RK designed the study with contributions from MS, OS, and KK. RK collected the data, with logistical support from OS. RK analyzed the data, with contributions from MS and KK. RK wrote the manuscript, with contributions from MS, OS, and KK. All authors contributed to the article and approved the submitted version.

## Funding

This research was carried out with the aid of grants from the International Development Research Centre, Ottawa, Canada (RK, grant number 106204–99906075-039) and the Natural Sciences and Engineering Research Council of Canada (RK, grant number PGSD3-392828-2010). The Panama Ministry of Health provided logistical and in-kind support for data collection activities and provided albendazole for treatment of all preschool children.

## Conflict of interest

The authors declare that the research was conducted in the absence of any commercial or financial relationships that could be construed as a potential conflict of interest.

## Publisher’s note

All claims expressed in this article are solely those of the authors and do not necessarily represent those of their affiliated organizations, or those of the publisher, the editors and the reviewers. Any product that may be evaluated in this article, or claim that may be made by its manufacturer, is not guaranteed or endorsed by the publisher.
